# Microvascular Angiopathic Consequences of COVID-19

**DOI:** 10.3389/fcvm.2021.636843

**Published:** 2021-02-02

**Authors:** Margaret Nalugo, Linda J. Schulte, Muhammad F. Masood, Mohamed A. Zayed

**Affiliations:** ^1^Section of Vascular Surgery, Department of Surgery, Washington University School of Medicine, St. Louis, MO, United States; ^2^Division of Cardiothoracic Surgery, Department of Surgery, Washington University School of Medicine, St. Louis, MO, United States; ^3^Division of Molecular Cell Biology, Washington University School of Medicine, St. Louis, MO, United States; ^4^Department of Biomedical Engineering, McKelvey School of Engineering, Washington University, St. Louis, MO, United States; ^5^Veterans Affairs St. Louis Health Care System, St. Louis, MO, United States

**Keywords:** COVID-19, micovascular disease, angiopathy, cardiac dysfunction, vascular thrombosis

## Abstract

The coronavirus disease-2019 (COVID-19) pandemic has rapidly spread across the world. The disease is caused by severe acute respiratory syndrome coronavirus 2 (SARS-CoV-2), which first appeared in Wuhan, China in December, 2019. Ever increasing data is continuing to emerge about the impact of COVID-19 on cardiovascular tissue and other organ system. Clinical features associated with COVID-19 suggest that endothelial cell dysfunction and microvascular thrombosis are to a large extent contributing to resultant multi-organ complications. This review is aimed at highlighting the critical aspects associated with COVID-19 and its presumed microvascular angiopathic consequences on the cardiovascular system leading to multi-organ dysfunction.

## Introduction

Coronaviruses (CoVs) are enveloped, non-segmented, single strand positive sense RNA viruses that are widely distributed among humans and other mammals. This group of viruses belongs to the coronaviridae family of viruses, and are harbored in animals such as civets, dogs, cats, bats, and camels (1–3). To-date there are six coronaviruses (CoVs) known to infect humans and these include; 229E, OC43, NL63, HKU1, Middle East Respiratory Syndrome Coronavirus (MERS-CoV), and severe acute respiratory syndrome coronavirus (SARS-CoV) (1, 3, 4). The 2002–2003 pandemic in Guangdong, Southern China was caused by SARS-CoV-1 (5), and resulted in >8,000 human infections, and 774 deaths in 37 countries (6). More recently in 2012 and 2015, MERS-CoV was responsible for the outbreaks that caused nearly 2,500 infections, and >850 deaths (7, 8). Although another CoV outbreak was predicted by various organizations, the scale of a resultant global pandemic was not completely anticipated.

The coronavirus disease-2019 (COVID-19) is a novel emerging infectious disease that is caused by SARS-CoV-2. COVID-19 was declared a global pandemic by the World Health Organization on March 11, 2020 (9). This viral disease first appeared in Wuhan, Hubei province, China in December 2019. According to the Johns Hopkins University Coronavirus resource center dashboard, as of September 2020 the disease was been confirmed in 188 countries, infected >26 million individuals, and caused >800,000 deaths world-wide (10). In the United States (US) alone there was >6 million confirmed cases, and >180,000 deaths (10).

Like other SARS-CoV, COVID-19 can cause a severe pulmonary viral pneumonia leading to severe hypoxia and subsequent respiratory failure. However, emerging evidence demonstrates that unlike the majority of upper respiratory viral illness, COVID-19 can also lead to cardiovascular and multi-organ complications (11–13). Although the basic biological understanding of these complications is still evolving and yet to be fully elucidated, there is emerging evidence that demonstrates that COVID-19 can cause microvascular angiopathy in various organ systems and tissue beds—including cardiovascular tissue (14, 15).

## Basic Biology

**Figure 1:**
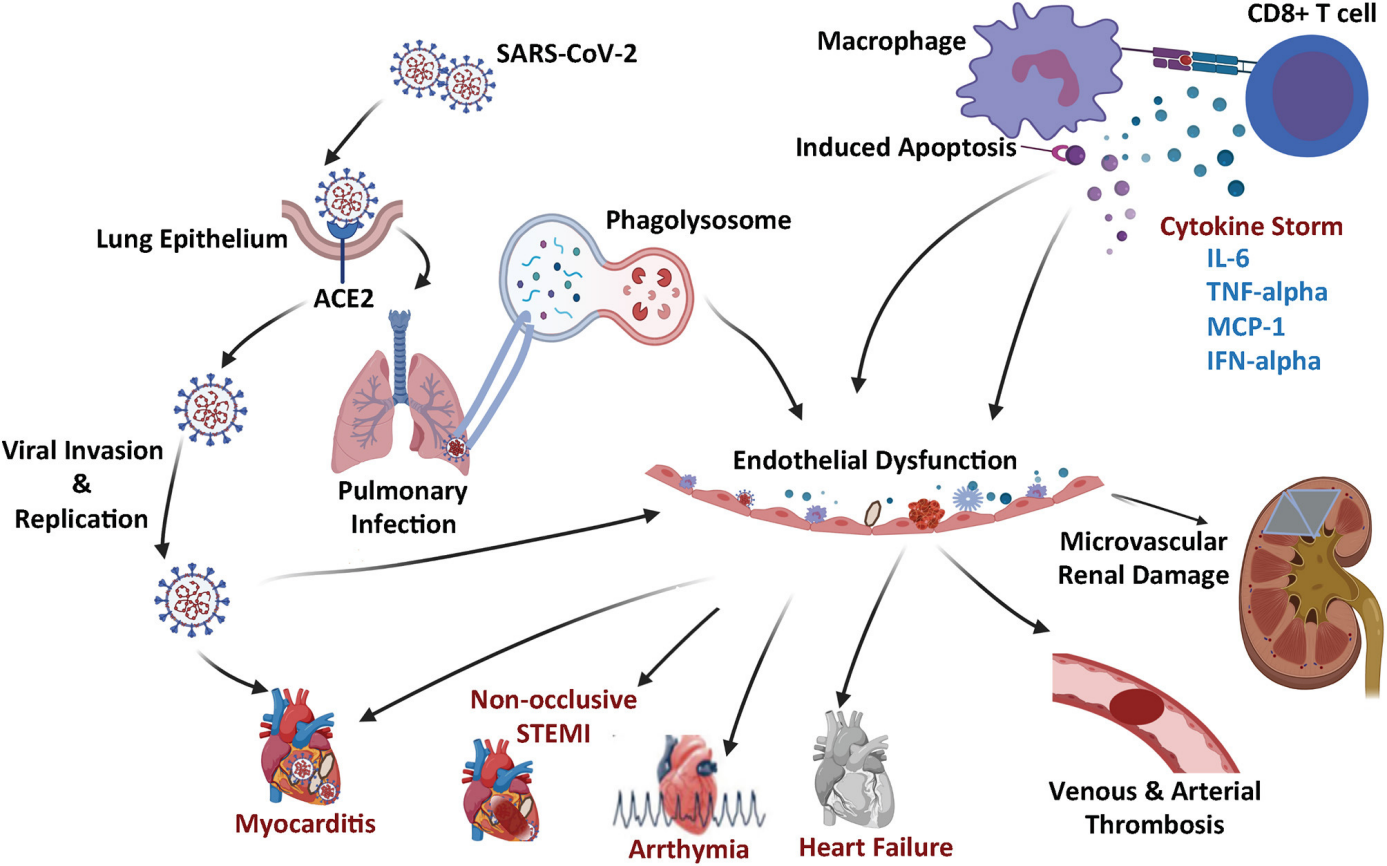
Schematic summary of the COVID-19 microangiopathic consequences leading to myocarditis, non-occlusive STEMI, arrhythmia, heart failure, vascular thrombosis, and microvascular renal dysfunction.

The SARS-CoV2 has been reported to bind to angiotensin converting enzyme 2 (ACE2) protein (16). Specifically, it was determined that the S1 domain of the SARS-CoV S protein can efficiently bind to the ACE protein on living cells. In these experiments, ACE2 antibodies were found to effectively block viral replication, but ACE1 antibodies did not have an impact on viral replication (16). Therefore, this data convincingly demonstrated that the ACE2 protein served as a critical attachment and entry vehicle for SARS-CoV2 viral particles into mammalian cells (Figure 1).

Upon binding onto the cell membrane ACE2 protein, the SARS-CoV2 virus enters the cell via a receptor mediated endocytosis and uses the host cell nuclear machinery to replicate (17), which produces multiples of virion replicates triggering an inflammatory response leading to the production of various cytokines, including but not limited to Interleukin-1beta (L-1β), interferon gamma (IFN-γ), and Monocyte Chemoattractant Protein-1 (MCP-1) (Figure 1). The chemokine (C-C mofit) ligand 2 (CCL2) also known as MCP-1 binds to its receptor on the surface of the microvascular ECs cells and pro-inflammatory macrophages (18, 19), further triggering more cytokine release and recruitment of additional inflammatory cells [such as monocytes, CD4 T helper cells, CD8 T cells, and Natural killer (NK) cells]. The resultant release of additional cytokines leads to a severe cytokine storm, which causes significant cellular and tissue damage (18).

The ACE2 mRNA is detectable in virtually all mammalian tissue, however, the protein is most remarkably expressed on the cell surface of lung alveolar epithelial cells and intestinal enterocytes (17). The protein is also highly expressed on the arterial and venous endothelium (17). Even prior to the COVID-19 pandemic, it was speculated that SARS-CoV2 viruses could potentially lead to vascular dysfunction, endothelial cell damage, and microvascular thrombosis given the relative abundance of ACE2 on the cell surface of endothelial cells (15, 20).

## Microvascular Angiopathy

Microvascular angiopathy is a pathological sequelae of a multitude of conditions that results from over-activation of host immune defense mechanisms. Patients impacted by sepsis or septic shock from a bacterial or viral infection, or autoimmune process, may fall victim to hyperimmune responses that can lead to significant host consequences (21–25). For example, dengue viral infections can lead an infected host to mount a hyperimmune response that results in activation of M1 macrophages and the release of pro-inflammatory cytokines such as TNFα, IL-1β, and IL-6 (23, 24). These pro-inflammatory mediators can directly act on the endothelium leading to acute cellular dysfunction, loss of endothelial barrier function and increased vascular leakage, and higher risk of microvascular thrombosis. As a consequence patients afflicted by dengue fever can sometimes present with clinical manifestations of multi-organ dysfunction, including acute respiratory distress syndrome (ARDS), myocarditis, acute kidney injury (AKI), and skin changes such as petechiae, bruising, and purpura (23).

Bacterial infections such as Lyme disease (*Borrelia burgdorferi*) and Rocky Mountain spotted fever (RMSF; caused by *Rickettsia rickettsii*) also can lead to severe cytokine storm, overexpression of CCL2, and resultant microvascular injury. In presence of such infections, patients can often develop microvascular dysfunction and thrombosis, as well as over-activation of the host fibrinolytic system (21, 22, 25, 26).

Prior literature establishes a clear interplay between inflammation, hypercoagulation, and thrombosis. Presumably part of this interplay is mediated by cytokine storm that leads to a higher propensity to development disseminated intravascular coagulopathy (DIC) (27). The systemic activation of the coagulation pathways by mediators generated during cytokine storm, can lead to a prothrombotic state characterized by the deposition of microthrombi, diffuse capillary obstruction, and the resultant tissue ischemia and organ damage. The tissue damage leads to further inflammation, and hence a vicious cycle ensues with additional inflammation leading to further coagulopathy, tissue damage, and increased risk of morbidity and mortality (27, 28). Current evidence estimates that >71% of patients who died from COVID-19 met the International Society on Thrombosis and Hemostasis (ISTH) criteria for DIC (29). Coagulation profiles for these individuals were typically notable for higher serum D-dimers, lower antithrombin III levels, and higher fibrin degradation products compared to healthy controls (30).

## Clinical Features

Since the emergence of the pandemic, there has been a multitude of clinical series published regarding the clinical manifestations and outcomes of COVID-19 (12, 31–34). The majority of the initial symptoms were related to the upper respiratory tract and gastrointestinal system, and varied significantly in severity between affected subjects. The common initial symptoms included; cough, dyspnea, fatigue, and myalgia (35). Additional minor symptoms included; headaches, and diarrhea (36).

The median incubation period has been reported to be 5 days and individuals who develop symptoms do so within approximately 11.5 days of infection (37). The median time from onset of initial symptoms to dyspnea is 8 days (38, 39). In some cases, the disease rapidly progressed to ARDS and septic shock, while in others, the disease took a milder course (38). Critically ill patients who required mechanical ventilation (15/21; 71%) typically presented with major multi-organ complications (31, 32, 40). These included non-atherosclerotic ST-elevation myocardial infarction, interstitial pulmonary tissue edema, AKI, brainstem infarcts, liver dysfunction, skin petechial rashes, and gastrointestinal mucosal bleeding—which were often associated with worse clinical outcomes (31).

## Organ System Complication

### Cardiac

**Figure 2:**
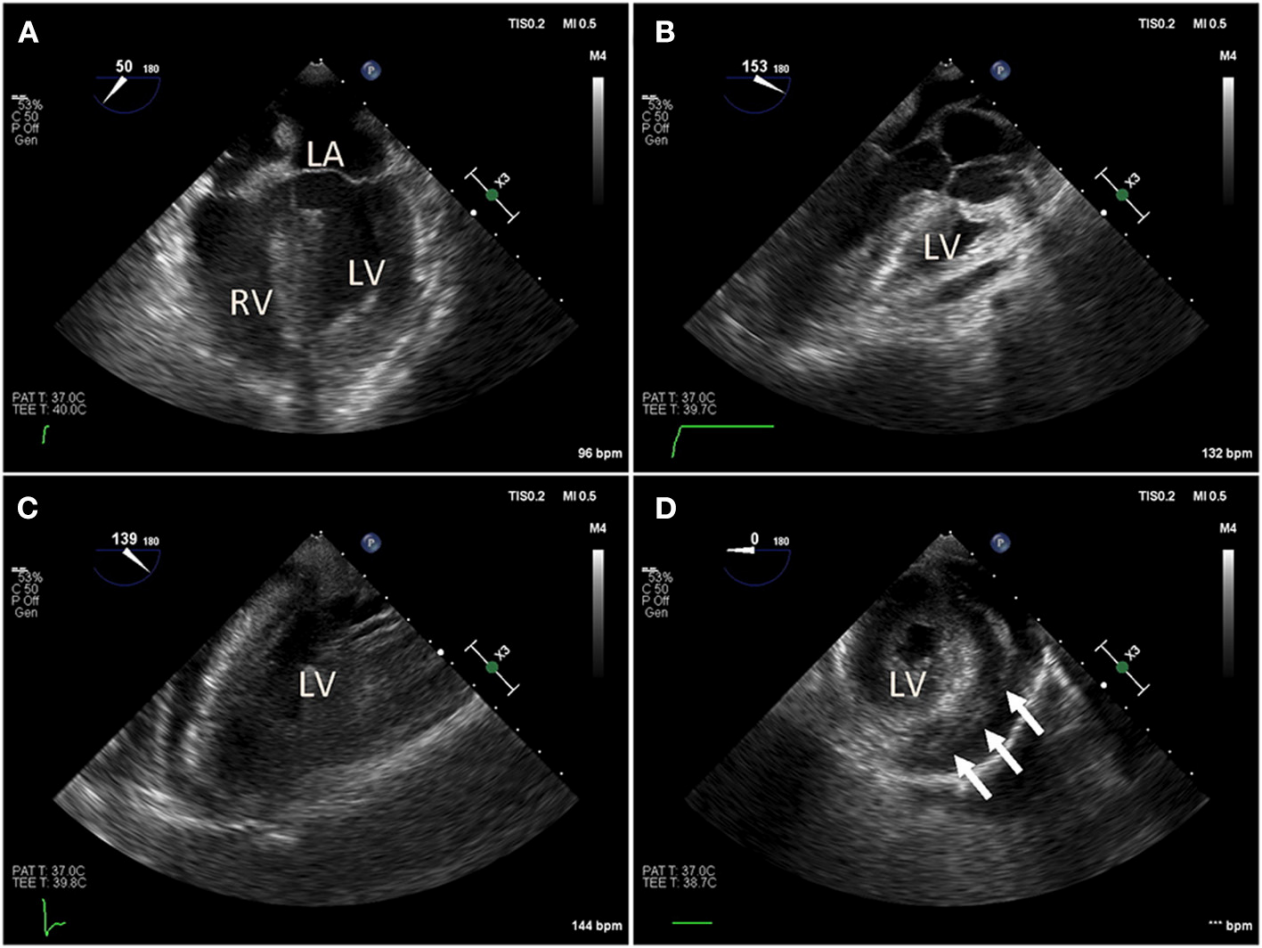
Intraoperative trans-esophageal echocardiography (TEE) in a COVID-19 positive patient with myocarditis, acute pericardial effusion and cardiac tamponade. After bedside venous-arterial extracorporeal membrane oxygenation (VA-ECMO) cannulation was performed, the patient was taken to the operating room emergently for ventral cardiac window exposure and decompression of the pericardial effusion. **(A)** Preoperative mid-esophageal four chamber view demonstrated severely reduced global left ventricle (LV) function. **(B,C)** Similarly, mid-esophageal long axis view demonstrated severely reduced LV contraction. **(D)** Transgastric short axis view demonstrated under-filling of the LV. Patient was taken emergently to the operating room for cardiac window decompression of the pericardial effusion and a large pericardial effusion. An Impella device is seen in the LV.

**Figure 3:**
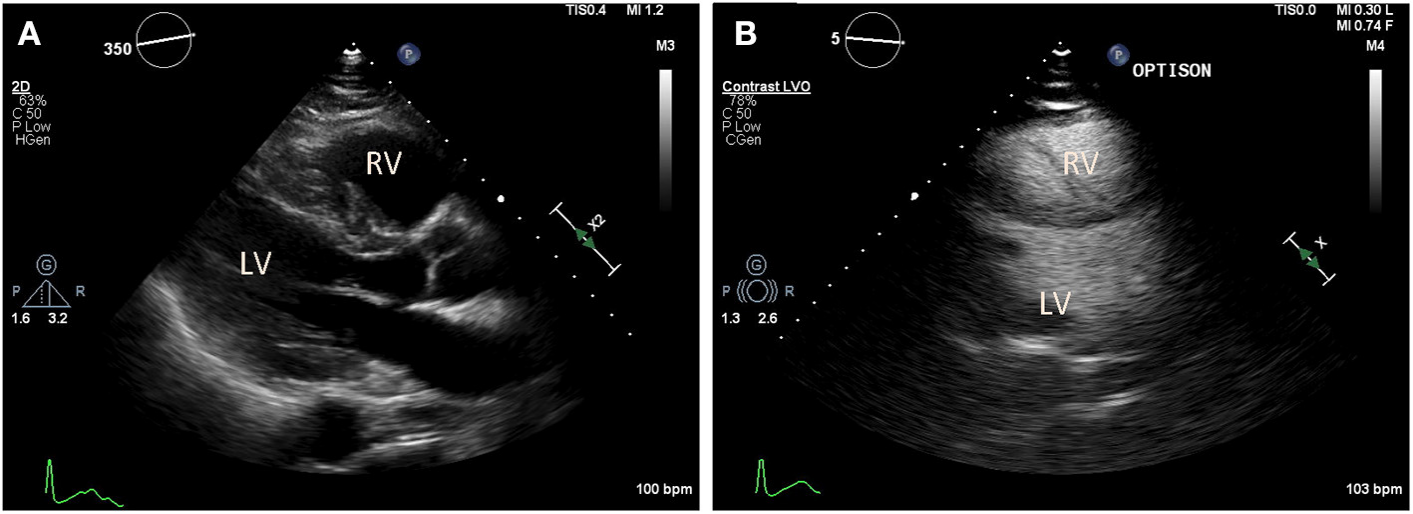
Transthoracic echocardiography (TTE) in a COVID-19 positive patient who presented with acute bilateral pulmonary emboli and acute right sided heart failure requiring emergent veno-venous extracorporeal membrane oxygenation (VV-ECMO) cannulation. **(A)** Parasternal long axis view demonstrate a McConnell's sign: Right ventricle (RV) enlargement and hypokinesis with preserved apical contractility. **(B)** Optison enhanced images demonstrate RV enlargement, and paradoxical septal motion consistent with RV dysfunction.

In addition to the known initial pulmonary consequences of COVID-19, various reports have demonstrated that patients infected with SARS-CoV2 can develop cardiac dysfunction and myocardial injury (41, 42). The mechanism of myocardial injury is unclear, but several have been proposed that severe respiratory dysfunction and consequential hypoxemia leads to increased myocardial demand, mixed severe respiratory and metabolic acidosis, which then results in myocardial injury and cellular apoptosis (12). Others propose that severe cytokine storm resulting from the host hyper-immune response to the primary SARS-CoV2 infection leads to secondary myocarditis, acute heart failure, malignant arrhythmias, and demand ischemia (Figures 2, 3) (43, 44). In addition, emerging reports suggest that SARS-CoV2 may act directly on myocardial tissue since ACE2 is presumed to be expressed on the myocardium with adequate quantities (41, 45). Primary viral myocarditis can occur with viruses such as cytomegalovirus (CMV) and Rubella but has not yet been confirmed with SARS-CoV infections (46–48). The occurrence of primary COVID-19 induced myocarditis would be a novel finding and may be consistent with recently reported echocardiographic findings that resemble other primary infectious myocardial complications such as Kawasaki syndrome (49, 50).

The incidence of acute cardiac injury in patients hospitalized with COVID-19 ranges between 7 and 17% (33, 35, 39). In one single-center series of 138 patients, individuals who were critically ill with COVID-19, were found to be more likely to have elevated myocardial injury serum biomarkers such as Troponin I and CK-MB (35). In another series of 120 patient hospital admissions due to COVID-19, 27.5 and 10% of the patients were found to have elevated levels of N-terminal pro B-type natriuretic peptide (NT-proBNP) and Troponin I, respectively (11). And, in another review of 187 patients with COVID-19, it was observed that 27.8% of patients who had myocardial injury and elevated cardiac serum biomarkers, had developed organic evidence of cardiac dysfunction and arrhythmias (51). Notably, in this cohort, patients with elevated serum Troponin I also had a 6.7 times higher odds of mortality. Non-occlusive STEMI is presumed to be the cause of cardiac enzyme release, suggesting that the primary cardiac pathology in the setting of COVID-19 may not be due to coronary artery thromboembolic obstruction (52, 53). The increased risk of mortality from cardiac factors also appears to be notably higher among individuals with baseline cardiovascular risk factors such as coronary heart disease, hypertension, and chronic cardiomyopathy (41, 51). Whether these baseline cardiovascular risk factors in some way are contributing to the primary COVID-19-induced pathology, rather than just serving as pre-disposing factors, is the subject of ongoing investigations.

Cardiac arrhythmias have also been reported as common complication in patients afflicted by COVID-19. One study of 121 patients, it was reported that >71% of patients experienced periods of sinus tachycardia, >14% of patients experienced bradycardia, and 1 patient developed new-onset transient atrial fibrillation (49). Other reports have cited development of malignant tachyarrhythmias is a strong predictor of mortality in patients infected with COVID-19 (11, 12, 35, 54). In one study the incidence of malignant ventricular arrhythmias was 75% more likely to be observed in patients with elevated serum Troponin (51). It is currently unclear whether these diverse types of arrhythmias are primarily a complication of COVID-19 infection itself, adverse effect of medication administered for treatment of COVID-19, or whether they are a result of exacerbation of the previously underlying heart disease.

Similarly, heart failure and fulminant myocarditis have been reported in a subset of patients infected with COVID-19. An analysis of 191 patients, reported that heart failure was observed in 23% of patients who were hospitalized due to COVID-19 infection. Patients with heart failure were noted to have an elevated (up to 28%) risk of mortality (33). Several reports also demonstrate clinical evidence of fulminant myocarditis with associated cardiogenic shock (42, 51). Preliminary evidence suggested that these conditions responded with hemodynamic improvement and stability following therapy with methylprednisolone, immunoglobulins, milrinone, and diuresis (42). It is not entirely clear whether resultant heart failure in these acute settings is an exacerbation of pre-existing heart disease or if it is due to the COVID-19 related myocarditis and stress-induced cardiomyopathy (55).

Various treatment modalities have been employed in the treatment of patients with COVID-19 and cardiac manifestations. Antivirals such as ribavirin, remdesivir, and oseltamivir, and antibiotics such as ceftriaxone, azithromycin, and immunoglobulins to modulate the immune status have also been reported with variable success (11, 49, 54). In addition, there have been reports on the utilization of invasive and non-invasive ventilation, extracorporeal membrane oxygenation, and intra-aortic balloon pump as rescue therapy maneuvers (11, 12, 35, 42).

### Brain and Neurologic

Various reports have cited evidence to suggest microvascular dysfunction in the central nervous system of patients with severe COVID-19 infections (56–60). The signs and symptoms associated with COVID-19 related brain dysfunction include; headache, seizures, anosmia and hypogeusia, impaired consciousness, ataxia, delirium, paralysis, and strokes (36, 56, 61, 62). A review of 214 patients from Wuhan, China, who were admitted to the hospital due to COVID-19 infections, demonstrated that 36.4% had neurologic manifestations. Patients with severe infection were more likely to have neurologic manifestations such as acute strokes (5.7%), and altered consciousness 14.8% (61). Brain computed tomographic (CT) scans confirmed the presence of large ischemic strokes in five patients, and one patient with intracranial hemorrhage (61). In another review of 219 Chinese patients, the authors also reported findings of acute ischemic stroke on head CT in 4.6% patients (63). Similar large ischemic stroke complications were also reported among patients treated in the New York Health System (64).

The ACE2 receptor is expressed on brain endothelial and smooth muscle cells (17, 56). Therefore, it is presumed that COVID-19 viral particles may be able to directly infect microvascular structures in the central nervous system, and lead to the observed neurological complications. This was confirmed by a report by Paniz-Mondolfi et al. (65) that demonstrated using transmission electron microscopy the presence of viral particles in small vesicles of brain endothelial cells. In another study involving 50 postmortem cases, a varied range of histopathological findings in the brain including focal and/or diffuse cortical, brainstem, and leptomeningeal inflammation (36%), encephalitis with localized perivascular and interstitial infiltrates with axonal degeneration and neuronal cell loss (12%), and inflammatory T-cell infiltrates with clusters of macrophages and axonal injury (66). These findings suggest that COVID-19-induced microvascular angiopathy can directly lead to brain and nerve tissue injury. It remains unclear which what patient populations are most prone to these complications, and what pre-disposing factors may lead to the more serious brain ischemic stroke manifestations.

### Liver

The ACE2 receptor is highly expressed in the gastrointestinal epithelium, and in liver cholangiocytes and arterial endothelium. However, it is not as highly expressed in the liver sinusoidal endothelium (67–69). A large subsets of patients infected with COVID-19 are noted to have liver pathology and transaminitis during the course of their hospitalizations (68–72). Reverse Transcriptase-Polymerase Chain Reaction (RT-PCR) has confirmed the presence of SARS-CoV genome in hepatic tissue, suggesting that COVID-19 may be directly binding to the liver microvasculature and causing hepatic complications (73, 74). Post-mortem studies of patients who died following COVID-19 infections demonstrated platelet-fibrin micro-thrombi within the hepatic microvasculature, various degrees of steatosis and portal histiocytic hyperplasia, and hepatic tissue ischemic necrosis (75). Another study reported patchy necrosis of the liver as well as wide infiltration by small lymphocytes (76). In a study of 10 postmortem examinations, the authors reported minimal periportal lymphoplasmacellular infiltration and significant fibrosis within the liver body (77). Although these studies have collectively examined a small subset of patients, they provide interesting insights into additional microangiopathic complications that may occur in the liver due to COVID-19 infection. Further studies may surely focus on potential disposing factors, or the contribution of underlying pathologies, in the resulting range of observed liver complications in patients who are acutely infected or chronically recovered from COVID-19.

### Renal

Initial reports from Wuhan, China informed us that AKI occurred in 7% of individuals who are COVID-19 positive (39). In patients who developed severe infection and required hospitalization, kidney abnormalities were observed in 25–50% of patients, and manifestations included acute proteinuria and hematuria (78). In a prospective cohort of 701 patients, from three different hospital centers in China, the authors observed that 43.9% of patients had proteinuria and 26.7% had hematuria, and were reported as adjusted risk factors for in-hospital death (79).

The ACE2 receptor is known to be expressed in the renal capillary endotheilum, and is thought to lead to localized tissue inflammation due to COVID-19 infections. Electron microscopy of renal capillary endothelium demonstrated COVID-19 viral inclusion structures, and on tissue histology inflammatory cells were visualized to be associated with endothelium—consistent with endothelitis (17). In a study reviewing renal histopathology from 26 patients who died following COVID-19 infections, there was histological evidence of diffuse proximal tubule injury, red blood cell aggregates obstructing capillary lumens, and clusters of viral particles in the tubular epithelium (80).

Several mechanisms of COVID-19 induced renal injury have been proposed. One potential mechanism may simply be due to hypo-perfusion from dehydration and diarrhea related to the systemic inflammatory (81–83). However, this mechanism would not necessarily explain findings of proteinuria and hematuria, which are not typically observed with other pre-renal conditions (83). An alternative hypothesis is that COVID-19 induced endothelial dysfunction may be disruptive to the vascular hemostatic equilibrium that shifts it toward a more vasoconstrictive state. This microangiopathic effect can lead to acute tissue malperfusion, ischemia, and a pro-coagulation state (84, 85).

The care of patients impacted by COVID-19 related AKI remains largely supportive. Depending on the severity of the presentation, patients may be managed with renal replacement therapy (RRT) in the form of intermittent hemodialysis or continuous renal replacement therapy (CRRT), or slow low efficiency dialysis (SLED). In the current absence of data clearly demonstrating superiority of one modality over another, the choice of dialysis modality is mostly informed by the availability of resources and local clinical expertise (82).

### Venous Thromboembolism (VTE)

**Figure 4:**
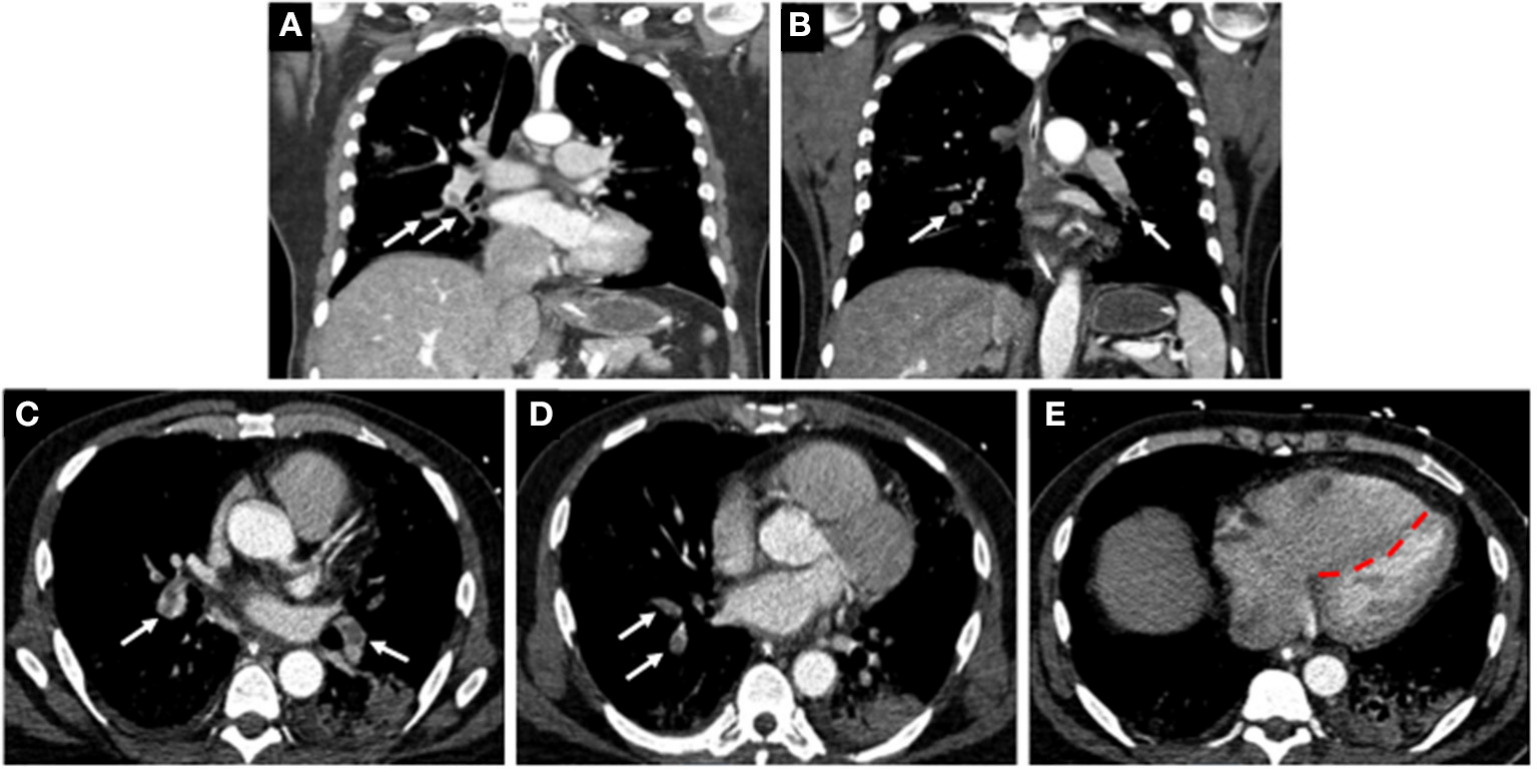
Computed tomographic (CT) images of a COVID-19 positive patient who presented with bilateral lobar pulmonary embolism (white arrows; **A–D**), and right ventricular enlargement and bowing of the interventricular septum to the left, consistent with right heart strain (red line; **E**).

Coagulopathy is a common phenomenon that can occur with sepsis and may predict outcomes in patients with severe pulmonary infections and multisystem organ dysfunction (86). Several reports indicate that patients with COVID-19 infections who are critically ill are at increased risk of developing VTE, including deep venous thrombosis (DVT) and pulmonary emboli (PE) (87–89). In a single center cohort study of 198 patients, 20% of patients were diagnosed with VTE and with extensive symptomatic thrombophlebitis for which anticoagulation treatment was initiated (90). The cumulative incidences of VTE at 7, 14, and 21 days was found to be 16, 33, and 42%, respectively (90). The authors also reported that VTE was significantly associated with death, with a hazard ratio of 2.7. In a separate single center study of 147 patients admitted to hospital for COVID-19, 17% were found to have VTE (91). Of these patients, 64% had acute PE, and 56% had acute DVT, and the all-cause mortality in these patients was significantly greater when compared to patients with no VTE (48% vs. 22%) (Figure 4) (91).

Some screening serum biomarkers have been proposed to monitor severity and incidence of VTE. In one study an admission D-dimer level >1,500 ng/mL was found to be an independent marker associated with the incidence of VTE (91). In another retrospective single center study with a total of 81 patients diagnosed with severe COVID-19 pneumonia admitted to the intensive care unit (ICU), D-dimer level >1,500 ng/mL again was a good index for identifying high risk groups (92). Similarly, in another study of 150 patients who were admitted to ICU due to COVID-19 infections, serum Von Willebrand (vWF) activity, vWF antigen, and FVIII were considerably increased, and 87.7% had positive lupus anticoagulant (93).

Multiple studies have demonstrated an overall higher index of morbidity and mortality in patients affected by VTE (94–96). For example, in a Dutch cohort of 184 patients who were admitted to ICU with COVID-19, the authors observed an adjusted cumulative incidence of symptomatic acute PE, DVT, ischemic stroke, myocardial infarction and/or systemic arterial embolism of 49% (96). Notably, the majority of the thrombotic events were PE (87%) in nature (96). Patients with thrombotic complications were at a higher risk of all-cause mortality (HR 5.4) and the use of therapeutic anticoagulation was associated with a 21% increased likelihood of survival (all-cause mortality HR 0.79) (96).

## Treatment Strategies

The treatment of COVID-19 associated microvascular angiopathies and their effects on the cardiovascular system is yet to be defined, but various strategies can potentially be derived from treatments used for management of other microvascular angiopathies. Conditions such as Kawasaki's disease, Henoch-Schonlein purpura, Rheumatoid Arthritis and Buerger's disease, are typically managed by targeting the underlying disorder and palliating symptoms (97–101). Some are vasculitidies responsive to treatment with immunoglobulins, calcium channel blockers, thrombolytics, prostaglandin analogs, glucocorticoids, and other immune-modulators such as methotrexate (97, 99, 101). Various such agents have been used in the battle against COVID-19 with variable success (12, 29, 89, 102–107).

### Anti-virals

On May 1st, 2020 the US Food and drug administration (FDA) issued an emergency use authorization of Remdesivir for adults and children with suspected or laboratory confirmed severe COVID-19 (102). Remdesivir binds and inhibits the RNA-dependent RNA polymerase that is essential for SARS-CoV2 viral replication (104). In a study of 61 patients with confirmed severe COVID-19 and evidence of hypoxia (56% receiving mechanical ventilation and 8% receiving veno-venous or veno-arterial extracorporeal bypass), at least one dose of remdesivir on a compassionate use basis, demonstrated clinical improvement and improved heart function in 68% of patients (108). Similarly, in a randomized double-blind placebo-controlled multicenter trial, comprising of 237 patients, the authors reported that remdesivir was not associated with statistically significant clinical benefits, however, the numerically faster time to clinical improvement requires confirmation in larger studies (109). Other antivirals like lopinavir and ritonavir (used for treatment of HIV), ribavirin (used for treatment of Hepatitis C), are under clinical investigation for the treatment of COVID-19 (102, 105, 110).

### Anti-coagulation

Anticoagulants have recently emerged as an important treatment modality for patients infected with COVID-19, and in particular patients who are considered higher risk individuals (111). Various institutional guidelines have recommended initiation of prophylactic or therapeutic anticoagulation in patients impacted by COVID-19 (112–115). In one retrospective study, it was observed that patients who received either prophylactic or therapeutic anticoagulation had lower incidence of myocardial dysfunction, mechanical ventilation, and in-hospital mortality (114). In another study, of 2,773 COVID-19 positive patients who were receiving mechanical ventilation, the hospital mortality rate was significantly reduced in patients that received therapeutic anticoagulation (62.7 vs. 29.1%) (116). Despite these clear advantages in the application of therapeutic anticoagulation in critically ill patients afflicted with COVID-19, there remains important unanswered questions about the role, duration of therapy, and potential consequences of anticoagulation therapy. It is also unclear whether anticoagulation therapy may be used to help in prophylaxis against COVID-19-induced multi-organ dysfunction.

### Steroids

Steroid therapy has been used for the treatment of severe ARDS due to COVID-19 infection (12). In a multi-center study of 213 patients with moderate to severe COVID-19 related symptoms, it was found that patient who were treated with methylprednisolone had a reduced incidence of escalation of care and improved clinical and cardiac outcomes (117). Similarly, in a multicenter partially randomized, preference open label trial, patients with a COVID-19 pneumonia, impaired gas exchange, and biochemical evidence of hyper-inflammation, demonstrated reduced incidence of death, cardiac dysfunction, ICU admission, and non-invasive ventilation following treatment with methylprednisolone (118). In another multicenter observational study, methylprednisolone administration in patients with severe COVID-19 pneumonia significantly lowered rates of ventilator dependence, myocardial infarction, and death (119).

## Conclusion

The body of evidence demonstrating the role of microvascular angiopathy in COVID-19 is evolving rapidly. It is evident from the current body of knowledge that COVID-19-induced microvascular angiopathy can lead to a wide range of tissue pathology and clinical complications. Understanding the molecular, biochemical and pathophysiological mechanisms underlying this process is paramount to our ongoing management efforts of individuals afflicted by COVID-19. Additional research is necessary to help further our understanding of microvascular angiopathic complications related to COVID-19, help develop novel therapeutics, and provide an effective cure to this new global pandemic.

## Author Contributions

All authors listed have made a substantial, direct and intellectual contribution to the work, and approved it for publication.

## Conflict of Interest

The authors declare that the research was conducted in the absence of any commercial or financial relationships that could be construed as a potential conflict of interest.
